# Supplementary material for: Intersection of TKS5 and FGD1/CDC42 signaling cascades directs the formation of invadopodia
Source: J Cell Biol. 2020 Jul 16;219(9):e201910132. doi: 10.1083/jcb.201910132 (PMC7480108; doi:10.1083/jcb.201910132)
Supplement: Table S2 — lists antibodies used in this study. [file JCB_201910132_TableS2.docx]

| Antibodies (use) | Type (species) | Supplier (Reference) |
| --- | --- | --- |
| FGD1 | Polyclonal  (rabbit) | Sigma (HPA000911) |
| Collagen type I cleavage site (Col1-^3/4^C) | Polyclonal  (rabbit) | ImmunoGlobe (0217-050) |
| Cortactin (IF) | Monoclonal (mouse) | Merck (clone 4F11, 05-180) |
| GAPDH (WB) | Polyclonal  (rabbit) | Santa Cruz (sc-25778) |
| GFP (IF) | Polyclonal  (chicken) | Abcam (ab13970) |
| GFP (WB) | Polyclonal  (Rabbit) | Abcam (ab6556) |
| MT1-MMP (MMP14) (IF, WB) | Monoclonal (mouse) | Merck (clone LEM-2/15.8, MAB3328) |
| TKS5 (SH3PXD2A) (IF, WB) | Polyclonal (rabbit) | Novus Biologicals ([NBP1-90454](https://www.novusbio.com/products/sh3pxd2a-antibody_nbp1-90454)) |
| TKS4 (SH3PXD2B) (IF, WB) | Polyclonal (rabbit) | Novus Biologicals (NBP1-93965) |
| SHIP2 (IF) | Monoclonal (rabbit) | Cell Signaling Technology (C76A7) |
| p130^CAS^ (IF) | Monoclonal (rabbit) | Cell Signaling Technology (E1L9H) |
| HRP-conjugated anti-rabbit IgG | Polyclonal (goat) | Sigma (A0545) |
| HRP-conjugated anti-mouse IgG | Polyclonal (goat) | Jackson ImmunoResearch (115-035-062) |
| Alexa Fluor 488 Phalloidin |  | Molecular Probes (A12379) |
| Alexa Fluor 546 Phalloidin |  | Molecular Probes (A22283) |
| Anti-rabbitAlexa488 | (Donkey) | Molecular Probes (A21206) |
| Anti-rabbit-Cy3 | (Donkey) | Jackson ImmunoResearch (711-165-152) |
| Anti-chickenAlexaFluor488 | (Goat) | Molecular Probes (A11039) |
| Anti-mouse-Cy3 | (Donkey) | Jackson ImmunoResearch (715-165-151) |
| Anti-mouseAlexa647 | (Donkey) | Molecular Probes (A31571) |
| GFP-Trap (IP) | nanobody | ChromoTek (gtma-20) |
| CellMask Deep Red Plasma membrane Stain |  | Molecular Probes (C10046) |

**Table S2. Antibodies used in this study.**

WB, Western blotting; IF, immunofluorescence; IP, immunoprecipitation.
