# Supplementary material for: Intersection of TKS5 and FGD1/CDC42 signaling cascades directs the formation of invadopodia
Source: J Cell Biol. 2020 Jul 16;219(9):e201910132. doi: 10.1083/jcb.201910132 (PMC7480108; doi:10.1083/jcb.201910132)
Supplement: Table S3 — lists siRNAs and plasmids used in this study. [file JCB_201910132_TableS3.docx]

**Table S3. siRNAs and plasmids used in this study.**

| Gene | Cat. No. or siRNA sequence (Sens) | Source |
| --- | --- | --- |
| siTKS5 ^a^ | 5’-ACAAUAACCUCAAAGAUGU-3’  5’-GGACGUAGCUGUGAAGAGA-3’  5’-CGACGGAACUCCUCCUUUA-3’  5’-GGAUAAGUUUCCCAUUGAA-3’ | Dharmacon |
| siTKS5-3’UTR ^a^ | 5’-CAGCACCGGTAGAGAAAGCAA-3’  5’-AGGCCTTTGGTTTGCGTCTTA-3’ | QIAGEN |
| siTKS4 ^a^ | 5’-GCGAAGACCAAGUCGACAU-3’  5’-GUGUUCAUCAGGCGAGAUA-3’  5’-GGACUGAGAUCUCGAGUUU-3’  5’-GAACAUAUGCCACGUUAAA-3’ | Dharmacon |
| siFGD1 ^a^ | 5’-GGACCACUCAAGACCGAUA-3’  5’-CCCAAGAGGUUGACAGUGA-3’  5’-UCUCAAGGACUAUCUGUUA-3’  5’-AUAAGCUGCUGAAGGUAUA-3’ | Dharmacon |
| siFGD1-3’UTR ^a^ | 5’-CAGCCAGTGTCCTTCACACGTA-3’  5’-CCCAGCTCAGTCAATACTTGA-3’ | QIAGEN |
| siMT1-MMP ^a^ | 5'-GGAUGGACACGGAGAAUUU-3',  5'-GGAAACAAGUACUACCGUU-3',  5'-GGUCUCAAAUGGCAACAUA-3',  5'-GAUCAAGGCCAAUGUUCGA-3' | Dharmacon |
| siCDC42 #04 ^b^ | SI00028413 (unknown sequence) | QIAGEN |
| siCDC42 #07 ^b^ | SI02757328 (unknown sequence) | QIAGEN |
| siNT (Non-Targeting) | 5’-UGGUUUACAUGUCGACUAA-3’ | Dharmacon |

^a^ Used as a mix. ^b^ Used individually.

| Plasmids | Source |
| --- | --- |
| TKS5-GFP (pEGFP-N1) | S. Courtneidge (OHSU, Portland) |
| TKS5-GFP/W861A | This study |
| TKS5-GFP/W1092A | This study |
| TKS5-GFP/W861A-W1092A | This study |
| TKS5-mCherry | S. Courtneidge (OHSU, Portland) |
| FGD1-GFP (pEGFP-C1) | E. Genot (INSERM, Bordeaux, France) |
| FGD1-ΔNTer (1-335)-GFP | E. Genot (INSERM, Bordeaux, France) |
| pET-MCN-His-GST-FGD1^18-335^ | E. Genot (INSERM, Bordeaux, France) |
| pET-MCN-His-GST-FGD1^335-717^ | E. Genot (INSERM, Bordeaux, France) |
| pET-MCN-His-GST-FGD1^717-920^ | E. Genot (INSERM, Bordeaux, France) |
| TKS4-GFP | A. Lányi (University of Debrecen, Hungary) |
| pEAK-Flag/INPP4B | Dr. L. Cantley (Harvard Medical School, Boston, MA) (Addgene # 24324) |
